# Proximal small bowel obstruction in a patient with cystic fibrosis: a case report

**DOI:** 10.1186/s40792-019-0701-y

**Published:** 2019-09-13

**Authors:** Zana Alattar, Caitlin Thornley, Milad Behbahaninia, Amy Sisley

**Affiliations:** 1 College of Medicine, University of Arizona College of Medicine - Phoenix, 550 E. Van Buren Street Phoenix, Phoenix, AZ 85004 USA; 2 Phoenix Integrated Surgical Residency, University of Arizona College of Medicine, 550 E. Van Buren Street Phoenix, Phoenix, AZ 85004 USA

**Keywords:** Cystic fibrosis, Abdominal pain, Small bowel obstruction, Distal intestinal obstruction syndrome, Surgical management

## Abstract

**Background:**

As advancements are made in the management of cystic fibrosis (CF), survival of the CF patient into adulthood has increased, leading to the discovery of previously unknown CF complications. Though gastrointestinal complications of CF, such as distal intestinal obstruction syndrome, are common, this case demonstrates a variant presentation of small bowel obstruction in this population.

**Case presentation:**

We describe a 42-year-old male with CF who presented with 2 days of worsening upper abdominal pain, emesis, and loss of bowel function. The patient had no history of any prior abdominal surgeries; however, imaging was concerning for high-grade mechanical small bowel obstruction possibly related to internal hernia. Given leukocytosis and diffusely tender abdomen found on further workup, the decision was made to proceed with diagnostic laparoscopy after a brief period of intravenous fluid resuscitation. Intraoperatively, the transition point was found in the mid-jejunum and was noted to be due to kinking of the bowel causing vascular congestion in the proximal portion. Surgical manipulation of the bowel was required for return of normal perfusion and patency.

**Conclusion:**

Though the exact mechanism cannot be definitively delineated, we speculate that the increased viscosity and prolonged intestinal transit time, characteristic of CF, resulted in inspissated fecal content in the proximal small bowel, which then acted as a lead point for obstruction. Thus, though small bowel obstruction in patients with CF is often attributed to distal intestinal obstruction syndrome, a broader differential must be considered. Early surgical intervention may be necessary to prevent bowel ischemia and subsequent small bowel resection in a patient presenting with concerning clinical and image findings, as was seen in this patient.

## Background

In the span of a decade (2000–2010), advancements in screening and management have led to a 1.8% yearly increase in survival of cystic fibrosis (CF) patients [1]. If the mortality rate continues along this trend, the projected median survival of CF patients born in 2010 is 56 years [1]. With a growing number of CF patients reaching adulthood, the medical community will be faced with unique complications previously unseen in this population. The following case discusses one such complication.

## Case presentation

A 42-year-old male with CF presented with 2 days of persistent, worsening bilateral upper abdominal pain, non-bloody non-bilious emesis, and constipation. His last bowel movement occurred approximately 36 h prior to presentation, despite the use of two enemas. The patient denied any prior history of similar abdominal pain and states his regimen of polyethylene glycol 3350 (Miralax) 2–3 times per week has normally maintained his bowel habits at one to two soft stools daily. Surgical history was negative for any prior abdominal surgeries. Review of systems was negative for symptoms concerning for malignancy.

On exam, vital signs were within normal limits. The patient was visibly uncomfortable and diaphoretic, with diffuse abdominal tenderness to palpation, worse in the left upper quadrant, mildly distended, though soft. There was no evidence of umbilical or inguinal hernias. Further workup revealed leukocytosis of 11,500 with left shift and normal lipase.

**Fig. 1:**
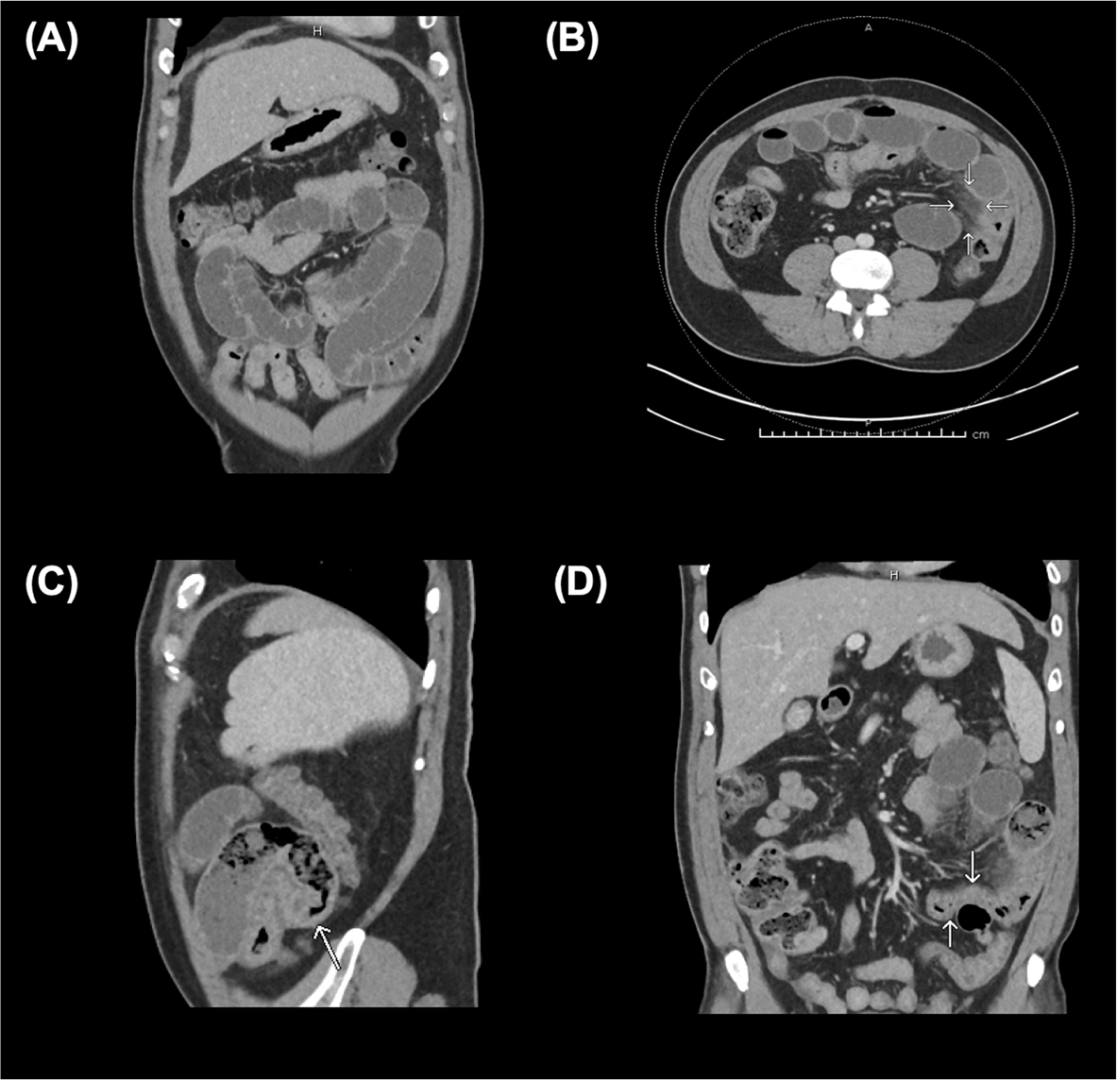
CT abdomen/pelvis with contrast. The CT scan of this CF patient demonstrated dilated loops of bowel proximally (**a**), with associated mesenteric venous congestion (**b**). Additionally, a transition point (**c**) was noted in the left hemiabdomen, distal to which the small bowel was decompressed (**d**). Just proximal to the transition point, gas bubbles and particulate matter were noted, suggestive of fecal contents in the small bowel

CT abdomen/pelvis with and without intravenous contrast demonstrated a high-grade mechanical small bowel obstruction, possibly related to internal hernia, with a transition point in the left lateral hemiabdomen (Fig. 1c) and inflamed mesentery surrounding proximally dilated small bowel (Fig. 1a, b).

Given the exam findings, leukocytosis, and high-grade mechanical obstruction with transition point found on imaging, the patient was taken to the operating room for exploration after a brief resuscitative period with intravenous fluids. Consents were obtained for possible bowel resection and/or ostomy and counseling on the possibility of encountering a mass/tumor.

The decision was made to proceed with a diagnostic laparoscopy, with low threshold for converting to laparotomy if visibility was an issue. Veress needle at the umbilicus was used to achieve pneumoperitoneum, and a 5-mm trocar was placed at the umbilicus using a camera through a clear trocar. Given good visibility, additional 5 mm trocars were placed in the right upper and right lower quadrants.

**Fig. 2:**
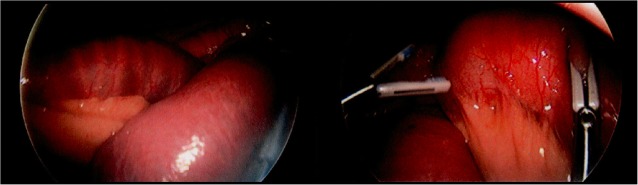
Dilated loops of bowel. Intraoperatively, hyperemia and dilation of the proximal small bowel was noted from the distal duodenum to the mid-jejunum. Hypervascularity of the bowel wall is suggestive of vascular congestion secondary to increased luminal pressures exceeding venous pressures. There was no evidence of ischemia, necrosis, or perforation

**Fig. 3:**
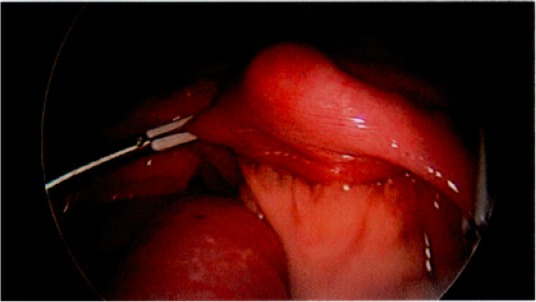
Transition point with decompressed loops of bowel distally. At the level of the mid-jejunum, kinking of the small bowel was noted, without involvement of the mesentery or evidence of adhesions. Immediately distal to this point, the bowel was entirely decompressed, without evidence of poor perfusion

The small bowel was ran from the ligament of Treitz to the ileocecal valve, revealing significant dilation and vascular congestion from the distal duodenum through the mid-jejunum (Fig. 2). There was a clear transition point in the mid-jejunum, where there was a kink (akin to a bascule) unrelated to a true volvulus or adhesive process (Fig. 3). Complete decompression of the small bowel distally was noted. Examination of the transition point suggested an extramural etiology secondary to intraluminal obstruction. This was relieved with external manipulation of the small bowel to achieve continuity and allow anterograde movement of bowel contents. With peristalsis, the distension and congestion of the proximal loops visibly improved. The bowel was observed until it returned to a well-perfused state. No resection was required. The surgery was concluded at this point. The patient tolerated the operation well, without complications.

Postoperative management included 12 h continuous polyethylene glycol solution at 100 cc/h via nasogastric tube. Following return of regular bowel function on postoperative day 2, he was discharged home with increased bowel care.

## Discussion

CF is an autosomal recessive disease caused by a heterogeneous spectrum of *CFTR* mutations resulting in deranged function of the cystic fibrosis transmembrane conductance regulator (CFTR). The lungs, pancreas, and intestines are usually affected by impaired secretion, absorption, and motility. In the gastrointestinal (GI) tract, reduced chloride and fluid secretion via the CFTR membrane protein, combined with enhanced sodium and fluid absorption via the epithelial sodium channels (ENaC), increases intraluminal viscosity [2]. Myenteric ganglionitis and leiomyositis of the gastrointestinal wall are thought to lead to impaired GI motility [3]. These impairments predispose to intestinal obstruction, most commonly distal intestinal obstruction syndrome (DIOS).

DIOS is characterized as an acute, complete or incomplete, intestinal obstruction of viscid fecal accumulation in the ileocecal region [2, 4]. It has an incidence of 23.3–35.5 episodes per 1000 patient years and a lifetime prevalence of 14–16% [5–7]. Patients typically present with acute abdominal pain, distention, and emesis, with a palpable right lower quadrant (RLQ) mass on exam [6]. Abdominal X-rays are significant for fecal loading in the RLQ, with air-fluid levels in the setting of a complete obstruction. CT scans demonstrate proximal small bowel dilatation with inspissated fecal material in the distal ileum [3]. Given pre-existing poor nutritional status, associated hypoalbuminemia, long-term corticosteroid use, and poor pulmonary function, the CF population are high-risk surgical candidates [7, 8]. Thus, medical management of DIOS is preferred [9]. The majority of DIOS are managed with oral rehydration therapy, stool softeners, and osmotic laxatives containing polyethylene glycol; prokinetic agents such as macrolide antibiotics and metoclopramide may also be considered [4, 7, 8]. In the setting of a complete obstruction, a nasogastric tube can be utilized and a gastrografin enema can be therapeutic [3, 7, 8]. Failure of medical management necessitates surgical intervention due to risk of bowel ischemia and perforation [9]. In a multi-center, comparative, retrospective study of 26 patients with 60 episodes of DIOS, 11 patients (18.3%) required surgical intervention [10].

**Fig. 4:**
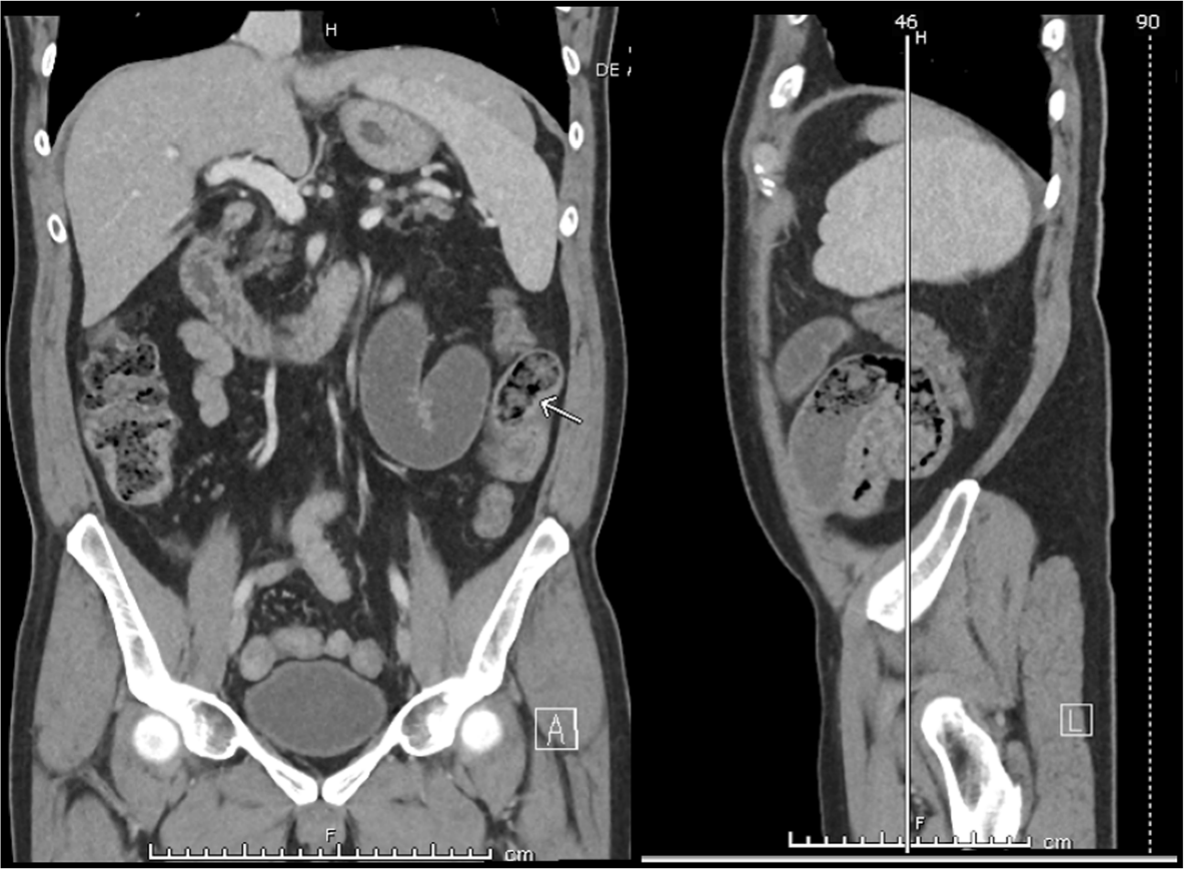
The small bowel feces sign. On further inspection, the patient’s CT scan was determined to have a characteristic small bowel feces sign in the mid-jejunum, just proximal to the suspected transition point. Defined in 1995 as the presence of gas and particulate material in a dilated segment of the small bowel, the small bowel feces sign indicates fecal content in the small bowel and is due to intraluminal stagnation of enteric material [11].

The case presented here represents a separate entity from DIOS owing to its unique location and mechanism of obstruction. The patient’s tenderness was localized to the LUQ, without significant tenderness or masses in the RLQ, as is common in DIOS. Rather than an ileocecal location (common in DIOS), CT imaging in our patient demonstrated a proximal obstruction on the left which was confirmed to be mid-jejunal intraoperatively. Additionally, the obstruction was noted to be mechanical—secondary to kinking of the bowel onto itself. Though the exact mechanism cannot be definitively delineated, we speculate that the increased viscosity of the intestinal contents and prolonged intestinal transit time, characteristic of CF, resulted in inspissated fecal content in the proximal small bowel (Fig. 4), which acted as a lead point for obstruction. As expected from the patient’s history, there was no evidence of adhesions, masses, or other lead points which could have caused the obstruction.

## Conclusion

Although small bowel obstruction in patients with CF is often attributed to DIOS and treated medically, a broader differential must be considered to determine appropriate management. Early surgical intervention may be necessary to prevent bowel ischemia and subsequent small bowel resection in a patient presenting with concerning clinical and image findings.

## Data Availability

All data generated or analyzed during this study are included in the published article.

## Abbreviations

CF: Cystic fibrosis
CFTR: Cystic fibrosis transmembrane conductance regulator
CT: Computed tomography
DIOS: Distal intestinal obstruction syndrome
ENaC: Epithelial sodium channels
ESPGHAN: European Society for Pediatric Gastroenterology, Hepatology, and Nutrition
LUQ: Left upper quadrant
RLQ: Right lower quadrant
SBO: Small bowel obstruction
WBC: White blood cell
